# Supplementary material for: When scientific experts come to be media stars: An evolutionary model tested by analysing coronavirus media coverage across Italian newspapers
Source: PLoS One. 2023 Apr 26;18(4):e0284841. doi: 10.1371/journal.pone.0284841 (PMC10132554; doi:10.1371/journal.pone.0284841)
Supplement: S1 File — (DOCX) [file pone.0284841.s001.docx]

### Supplementary Information for “When scientific experts come to be media stars: an evolutionary model tested by analysing coronavirus media coverage across Italian newspapers”

### TIPS project Named Entity Resolution and Entity Linking procedure for counting scientists mentions in newspaper articles

TIPS platform has developed a Named Entity Resolution and Entity Linking (N.E.R./E.L.) pipeline written in Python aimed to detect entities linked only to people, and specifically those engaged in scientific research, being mentioned in newspaper articles. This procedure addresses different orders of issues: (1) *epistemic* issue: what do we count as a “scientist”? We built a list of professional labels that are linked to entities, as “violinist”, “virologist”, and we discriminate scientists according to those, along with the number of scientific articles being authored under the entity name. (2) *Linking only people* issue: since schools, streets, research centers are often named after scientists, we should discriminate when the scientist name is not linked to scientists but to artifacts named after them. We do so looking for specific strings immediately preceding or following of the entity. (3) *Disambiguation*, or being able to discriminate aliases, homonyms and other ambiguities. Homonyms: the same name may be linked to several different persons. We should link the correct entity and avoid both homonyms with non-scientists (eg: “Paolo Rossi” may be linked, among others, to two soccer players, a comedian, an historian of science and many researchers) and homonyms with other scientists with the same name. We try to do so inspecting the context looking for semantic cues that may help to include/exclude the entity in counts. Aliases: the same entity may be written in different ways, or spelled differently according to languages, be misspelled, nevertheless we should link the same entity. Moreover we should consider that very famous scientists are often named by surname only (as “Newton” links to “Isaac Newton”). Other ambiguities**:** some scientists are also musicians or politicians, may figure also as actors in movies or be engaged and excel in sports. We should try to count them only when they are named as scientists. We do so by inspecting the context of the article and looking for semantic cues (specific words). (4) *Early detection*: while many resources are available online for Entity Linking (E.L.), WikiData being one among the richest and widely used, we could not entirely rely on them because newspapers may mention people not yet known to the public and not yet available in general E.L. platforms. We address this issue combining use of general E.L. resources with resources that link specifically scientists as PubMed, ORCID, Scopus.

We used a multi-stage approach, combining multiple tools and automating as much as possible each step, but closely inspecting results in order to build a knowledge base links entities and helps to inspect results to detect errors.

The pipeline steps detailed below are: 1) detect candidate entities → 2) filter people entities → 3) link to scientists → 4) exclude toponyms → 5) detect ambiguities and count only entities referring to scientists.

In detail:

1) build a list of candidate entities (NEC), combining the results of the Spacy library for Named Entity Recognition and a specific Regular Expression to catch people’s names (groups of two or more consecutive words of at least two letters, each beginning with an upper case character, separated by spaces). At this step we are building the list of entities, so false negatives (erroneous rejections) are tolerable: on the total number of articles, it is very unlikely that a name gets systematically rejected. On the contrary, false negatives (accepting strings as names when they aren’t) could bring in much more noise. Combining Spacy and Regular expressions gives a result which is accurate enough^[[1]](#footnote-1)^. At a later stage we will provide exceptions for very famous scientists who are usually cited only by last name (‘Einstein’, ‘Newton’) or first name (‘Galileo’).

2) Select only entities related to people: a layered approach selects only a candidate entity whose type is likely “person”: we did this with a lexical approach comparing the first portion of the entity to a number of *lists of first names* obtained by the following sources:

- 18957 names from Wikipedia APIs, Category:Given_names

- 19948 names from <https://github.com/smashew/NameDatabases>

- 23355 names from <https://github.com/alodish/BTN-name-extract>

- 552 names from TIPS project’s own supplement list.

While many names from the first three lists overlap, a stoplist (185 items) excludes names that often give false positives. This step selects roughly half of the entity candidates, while the other half is rejected. The supplement list is updated upon manual inspection of rejected first names.

At this stage, entities are not yet linked: misspelled names or different combinations of names figure as different named entities. Manual inspection highlights the frequent misspellings present in newspaper articles, which are noted to allow disambiguation and correct linking at a later stage.

3) Link entities to scientists with a layered approach in which each entity is searched in a cache of already found Named Entities (it’s a “Known” entity), by querying sources of generic knowledge as Wikipedia/Wikidata and of specific knowledge as Scopus (elsapy library^[[2]](#footnote-2)^), Orcid (https://pub.orcid.org/v3.0/expanded-search/), Imdb (imdb library^[[3]](#footnote-3)^), WoS (https://wos-api.clarivate.com/api/woslite), Pubmed (Bio/Entrez^[[4]](#footnote-4)^). Wikipedia description label is used in the forst place to build a list of professions/occupations that we accept and label as “scientist” ones for our research purpose; each label is tagged consequently as ‘PER’ for person, and ‘SCI’ for scientist: e.g. “virologist” → PER,SCI; “violinist” → PER.

According to results from queries in cache, generic and specific sources, a vector is built of ternary values (True/False/None) following matches found: [known (True/None), scientist (True/False/None), person (True/False/None), publications (if at least two of WOS>5 or Pubmed>5 or SCOPUS >5: True, otherwise: None), orcid (ORCID > 0: True, otherwise: None), celebrity (IMDB > 0: True, otherwise: None)].

Following a manually constructed matrix of all occurring combinations of values, entities are automatically classified alternatively as: NONPER: non-person, PER: person, MAYPER: maybe person, SCI: scientist, MAYSCI: maybe scientist, SCI: scientist, UNK: unknown.

If the specific combination is not already present in the matrix, an exception is raised and manually coded.

For all “MAYSCI” and “MAYPER” labeled entities we manually inspect the context of the 20 words surrounding the entity in newspaper articles and label them consequently. Similarly we look for errors for other labels . We limit inspection to entities mentioned in at least five articles in all the corpus over a year.

At the and of this phase each entity gets a NONPER, PER or sci SCI label either automatically or manually through a computer-assisted process, and kept in cache as a “Known” entity.

At this point we are ready to count the occurrence of each entity in newspaper articles, taking into account the issues above (avoiding toponyms, aliases, homonyms and other ambiguities):

4) Filter toponyms and named artifacts: entities that immediately are preceded or followed by terms that are specific to toponyms or institutions, as ‘via’, ‘scuola’, ‘istituto’, ‘lago’, ‘Nord’, etc. are automatically excluded.

5) Each entity labeled as “SCI” (scientist) is associated with lists of terms that, if present in the same sentence of the newspaper article, helps to resolve ambiguities and link to unique entities.

- Short names: i.e. “Galileo” for “Galileo Galilei”

- Aliases: i.e. “Levi Montalcini” for “Rita Levi-Montalcini”

- AND-terms: specific terms that must be in the same sentence of the named entity to give a positive count: i.e. “Susan Hopkins” AND ‘epidemiologist epidemiologa’

- NOT-terms: specific terms that should not be in the same sentence to give a positive count: “James Webb” NOT ‘telescope Telescope telescopio Telescopio JWT’

Those lists of terms, combined as in “Gabriele Riccardi”AND professore NOT ‘artista, pittore, scultore, cinquecento’ help to discriminate homonyms so that only articles about the professor are counted and not those about the painter under the same name.

Manual inspection of the entities both counted and excluded in the context of the sentence helps to refine this step until no attribution errors are detected. At this point the occurrences of entities labeled as SCI (scientist) in articles are counted.

1. For spacy, see <https://spacy.io/> . For a combined approach and NER tool rankings, see: Jiang, Ridong, Rafael E. Banchs, and Haizhou Li. "Evaluating and combining name entity recognition systems." *Proceedings of the Sixth Named Entity Workshop*. 2016. [↑](#footnote-ref-1)
2. <https://github.com/ElsevierDev/elsapy>, [↑](#footnote-ref-2)
3. <https://pypi.org/project/IMDbPY/> now CinemaGoer [↑](#footnote-ref-3)
4. <https://pypi.org/project/pymed/> [↑](#footnote-ref-4)
