# Supplementary material for: When scientific experts come to be media stars: An evolutionary model tested by analysing coronavirus media coverage across Italian newspapers
Source: PLoS One. 2023 Apr 26;18(4):e0284841. doi: 10.1371/journal.pone.0284841 (PMC10132554; doi:10.1371/journal.pone.0284841)
Supplement: Attachment — Submitted filename: response_PLOS ONE_WEMS.docx [file pone.0284841.s002.docx]

10 March 2023

Dear Reviewers and Assigned Academic Editor,

thank you for the opportunity to revise and resubmit the manuscript “When scientific experts come to be media stars: an evolutionary model tested by analysing coronavirus media coverage across Italian newspapers” to be considered for publication as a research article in PLOS ONE.

The reviewers’ suggestions were highly insightful and enabled us to improve the quality of our manuscript.

We believe we came across a solution able to address all the main issues and concerns raised by the reviewers. Accordingly, we integrated the required revisions.

About **data availability**, we made the “General Corpus A1” publicly available. We provided all information necessary for interested researchers to gain access to the data: URLs (the URLs where the article was published), sources (newspaper and feed/section where the article was published), datesPublished (dates when the article was published/updated). This dataset is adequate to replicate our study findings, and it has been deposited in a public repository, Zenodo, under the following doi: [10.5281/zenodo.7712714](https://doi.org/10.5281/zenodo.7712714) (Data from: When scientific experts come to be media stars: an evolutionary model tested by analysing coronavirus media coverage across Italian newspapers).

Below you can find our point-by-point responses to the major comments from and concerns of the reviewers and assigned academic editor.

- As required by both **rev.#1** and **rev.#2**, the possible relationships between visibility in daily newspapers and in TV or social media has been discussed both within the 4.1 session and in the concluding remarks where limits and significance of testing the proposed MEEM model only in newspapers are discussed. It has been also clarified that the research did not collect data regarding TV and/or social media, but it is possible to argue that what is observed in the newspapers can also offers indications about the presence of scientists in other media.
- As required by **rev.#1** and **rev.#2**, some hints about disciplinary fields and potential readership/scholars have been added in the conclusion.
- About other requests by **rev.#1** :

*- Please consider reviewing the abstract, which sounds a bit repetitive in some of its parts* ⇒

the abstract has been reviewed, also deleting redundant passages;

- *Line 42: Consider starting a new paragraph* ⇒ done;

- *Line 340: Regarding the nine periods into which the pandemic emergency in Italy has been divided, is it possible to cite an official source (perhaps through a link or website) or have the authors simply and independently relied on the dates of the Decrees-Laws promulgated by the Italian government?* ⇒ it has been added the link to the Italian Ministry of Health where all the official documents, decrees and rules are listed.

- About other requests by **rev.#2** :

*- pag. 23: As for Lopalco, as you probably know, at the end of 2020 he had a political charge as Assessor for Welfare in Puglia. Maybe this factor also influenced his "losings" in media; on the other side, this information could take a reflection about how being a "star" can help to gain new opportunities in other life domains* ⇒ a brief comment concerning the media trajectory of Lopalco has been inserted in 4.2 session.

Once again, our grateful thanks for your care and attention. We hope that we have made good use of your efforts.

The authors
